# Neural correlates of emotional processing in trauma-related narratives

**DOI:** 10.1017/S0033291724003398

**Published:** 2025-02-11

**Authors:** Minne Cao, Shengnan Zhu, Enze Tang, Chuang Xue, Kun Li, Hua Yu, Tao Zhong, Tao Li, Hui Chen, Wei Deng

**Affiliations:** 1 Affiliated Mental Health Center & Hangzhou Seventh People’s Hospital, Zhejiang University School of Medicine, Hangzhou, Zhejiang, China; 2 Department of Psychology and Behavioral Sciences, Zhejiang University, China; 3 Shandong Daizhuang Hospital, Jining, China; 4 Liangzhu Laboratory, MOE Frontier Science Center for Brain Science and Brain–Machine Integration, State Key Laboratory of Brain–Machine Intelligence, Zhejiang University, Hangzhou, China; 5 NHC and CAMS Key Laboratory of Medical Neurobiology, Zhejiang University, Hangzhou, China

**Keywords:** explicit declaration, functional near-infrared spectroscopy, implicit fear memory, post-traumatic stress disorder

## Abstract

**Background:**

Post-traumatic stress disorder (PTSD) is a mental health condition caused by the dysregulation or overgeneralization of memories related to traumatic events. Investigating the interplay between explicit narrative and implicit emotional memory contributes to a better understanding of the mechanisms underlying PTSD.

**Methods:**

This case–control study focused on two groups: unmedicated patients with PTSD and a trauma-exposed control (TEC) group who did not develop PTSD. Experiments included real-time measurements of blood oxygenation changes using functional near-infrared spectroscopy during trauma narration and processing of emotional and linguistic data through natural language processing (NLP).

**Results:**

Real-time fNIRS monitoring showed that PTSD patients (mean [SD] Oxy-Hb activation, 0.153 [0.084], 95% CI 0.124 to 0.182) had significantly higher brain activity in the left anterior medial prefrontal cortex (L-amPFC) within 10 s after expressing negative emotional words compared with the control group (0.047 [0.026], 95% CI 0.038 to 0.056; *p* < 0.001). In the control group, there was a significant time-series correlation between the use of negative emotional memory words and activation of the L-amPFC (latency 3.82 s, slope = 0.0067, peak value = 0.184, difference = 0.273; Spearman’s *r* = 0.727, *p* < 0.001). In contrast, the left anterior cingulate prefrontal cortex of PTSD patients remained in a state of high activation (peak value = 0.153, difference = 0.084) with no apparent latency period.

**Conclusions:**

PTSD patients display overactivity in pathways associated with rapid emotional responses and diminished regulation in cognitive processing areas. Interventions targeting these pathways may alleviate symptoms of PTSD.

## Introduction

Post-traumatic stress disorder (PTSD) is a psychiatric disorder stemming from the dysregulation or overgeneralization of memories associated with traumatic events, characterized clinically by symptoms of avoidance, intrusive experiences, and heightened alertness linked to fear memories (Harrison et al., 2018). Epidemiological studies have shown that not all individuals exposed to similarly intense traumatic events develop PTSD. The lifetime prevalence of PTSD is approximately 7.8% (Wisco et al., 2014), with a 12-month prevalence ranging between 1.3% and 3.6% (Wisco et al., 2016), indicating variability in the processing of fear memories among individuals(Harrison et al., 2018). Traumatic incidents elicit strong emotional reactions such as anger, fear, sadness, and shame, which can be reflected through language (Krüger-Gottschalk et al., 2022). Miragoli and Jennings’s works have indicated that individuals with PTSD use negative and neutral emotional words more frequently than those without PTSD (Jennings et al., 2021; Miragoli et al., 2014). Additionally, some individuals may try to avoid emotional outbursts when confronted with traumatic memories, leading to emotional numbness or the use of substances to dodge unwanted emotions (Brem et al., 2018). Thus, understanding the synergistic role of explicit narrative and implicit emotional memory in the processing of traumatic events is crucial for revealing the mechanisms underlying PTSD formation.

Patients with PTSD exhibit certain characteristics in their descriptions of traumatic events, including slower, more monotonous speech with less variation in tone and activation (Marmar et al., 2019). These features reflect the difficulties they face in processing emotional information. Specifically, this language pattern may be influenced by emotional states, agitation, and anxiety levels (Marmar et al., 2019), illustrating the impact of implicit emotional memory on explicit narrative. Greater use of bodily sensations, perceptual details, and negative emotional words (Ng et al., 2015), as well as frequent use of singular pronouns (D’Andrea et al., 2012) and death-related terms in trauma narratives (Papini et al., 2015), reflect individuals’ emotional responses during trauma such as fear, helplessness, horror, guilt, and shame (Kleim et al., 2018; Ozer et al., 2003). These features represent not just surface-level descriptions of traumatic events but are outcomes of deep emotional processing. Therefore, it can be posited that explicit narratives are the result of processing traumatic events, influenced significantly by implicit emotional memories, and these two aspects interact synergistically.

At the neural level, studies have explored PTSD’s neural correlates by activating traumatic memories through script-driven imagery. Exposure to traumatic scripts results in decreased functional connectivity between the medial prefrontal cortex (mPFC) and the amygdala (Shin et al., 2004). The anterior cingulate cortex, frontal lobes, and thalamus are also implicated in PTSD neural circuits (Lanius et al., 2001). Additionally, PTSD patients may exhibit functional dysregulation in the mPFC, hippocampus, and visual association cortex (Bremner et al., 1999; Rauch, 1996), as well as reduced functional connectivity strength in the prefrontal-limbic-thalamic-cerebellar circuitry (He et al., 2022). These findings suggest abnormal top-down emotional regulation processes in PTSD patients when facing traumatic events.

Notably, studies in healthy populations have shown that contextual processing, semantic tasks, and memory retrieval activate the anterior mPFC (amPFC), as well as the hippocampus and parahippocampal gyrus (Foudil & Macaluso, 2024; Lee & McCarthy, 2015). Activation in these regions is closely associated with episodic memory and emotional processing (Brown et al., 2018), indicating the amPFC’s significant role in processing traumatic events. It is speculated that PTSD patients may exhibit functional abnormalities in these areas, affecting emotional and memory processing.

According to implicit emotional learning theory, top-down regulation involves two pathways: the low pathway offers a fast but imprecise emotional response route, while the high pathway entails complex perceptual and cognitive processing, leading to more refined and adaptive emotional responses (Gazzaniga & Mangun, 2014). Typically, trauma-related fear memories are first encoded in the amygdala, then integrated and consolidated in the hippocampus (Albo & Gräff, 2018; Dudai, 2004; Frankland & Bontempi, 2005; Goto et al., 2021; Kitamura et al., 2017), and ultimately achieve long-term storage in the prefrontal cortex (Goto et al., 2021; Sun et al., 2020). The mPFC plays a crucial role in the maintenance, expression, and extinction of fear memory by coordinating emotional responses through the regulation of network connections with the amygdala (Karalis et al., 2016). However, PTSD patients may experience dysregulation in this process (Barkay et al., 2012), resulting in decreased cognitive control over negative emotions and an intensified experience of negative emotions (He et al., 2022).

To further investigate the regulatory mechanisms of implicit emotional memory during explicit narrative processes in PTSD patients, this study focuses on the amPFC as the region of interest. We employ functional near-infrared spectroscopy (fNIRS) to measure real-time brain activation levels during self-recalled trauma narratives. Additionally, we analyze narrative content characteristics to explore the relationship between explicit narratives and implicit emotional memory.

fNIRS offers high temporal resolution and tolerance to motion artifacts, making it suitable for immediate brain activity detection during language tasks (Curtin et al., 2019; Mazziotti et al., 2022). Moreover, fNIRS operates silently, avoiding environmental noise interference with speech tasks and recordings (Hagenaar et al., 2024), aligning well with the design requirements of this study.

Based on this background, the following hypotheses are proposed: (1) during trauma recall, PTSD patients rely more on explicit narratives of negative emotional memories and experience greater emotional distress during expression compared with the control group. (2) There is a time-series correlation between content features of explicit narratives (such as the use of negative emotional memory words) and activation of the amPFC. (3) PTSD patients exhibit sustained high activation of the amPFC during trauma recall narratives, particularly when expressing negative emotions, with higher activation levels than those in the control group.

## Methods

### Participants

An a priori power analysis was performed using G*Power 3.1 to determine the required sample size for the study (Faul et al., 2007). Anticipating an effect size of Cohen’s *d* = 0.7—based on previous studies involving neuroimaging and language measures between patient and control groups—and setting a significance level (*α*) of 0.05 and power (1 − *β*) of 0.80 for a two-tailed test, the analysis indicated that at least 34 participants were needed per group (Brown et al., 2018; De Boer et al., 2020). Accordingly, we recruited 35 patients with PTSD and 37 trauma-exposed controls (TEC).

All participants had experienced traumatic events conforming to the criteria of the Clinician-Administered PTSD Scale for DSM-5 (CAPS-5) (Weathers et al., 2015). The PTSD group consisted of 35 unmedicated patients diagnosed by professional physicians according to DSM-5 standards, with CAPS-5 scores equal to or exceeding 45 points. The TEC group included 37 individuals who were trauma-exposed according to DSM-5 PTSD criteria but did not meet any Axis I DSM-5 diagnoses. All participants underwent the same experimental procedures.

Eligibility criteria for participation included: (1) age 18–60; (2) right-handed; (3) native Mandarin speakers; (4) normal hearing and vision, or corrected to normal; (5) no history of brain trauma, epilepsy, or other organic brain disorders. Participants were excluded if they met any of the following criteria: (1) history of head trauma; the presence of organic brain diseases, neurological disorders, or severe endocrine or metabolic diseases; (2) diagnosis of other mental disorders according to DSM-5 criteria, including schizophrenia spectrum and other psychotic disorders, bipolar and related disorders, depressive disorders, anxiety disorders, obsessive–compulsive and related disorders, somatic symptom and related disorders, among others; (3) any severe neurological diseases or impairments, including but not limited to conditions associated with increased intracranial pressure, space-occupying brain lesions, history of epileptic seizures, cerebral aneurysm, Parkinson’s disease, Huntington’s disease, multiple sclerosis, or severe head trauma accompanied by loss of consciousness; (4) alcohol or substance dependence within the past 6 months; (5) received modified electroconvulsive therapy within the past 6 months; (6) pregnancy.

This study was approved by the Affiliated Mental Health Center & Hangzhou Seventh People’s Hospital, Zhejiang University School of Medicine Ethics Committee (Approval numbers: [2022] Ethical Review No. [041] and No. [064]), adhering to the Declaration of Helsinki. All participants signed an informed consent form after fully understanding the study details. The recruitment period was from August 2022 to January 2024.

### Data acquisition

Data were acquired using a 48-channel near-infrared optical imaging system (NirScan, Danyang Huichuang Medical Equipment Co., Ltd., China), covering the bilateral prefrontal cortex (PFC) and temporal lobes. The sampling rate was set at 11 Hz, with wavelengths of 730, 808, and 850 nm (730 and 850 nm being the primary measurement wavelengths, 808 nm for isotope correction). Based on the international 10–20 electroencephalogram system, 15 sources and 16 detectors were configured. To ensure adequate coverage of the target brain regions, the detector patches were placed vertically 3 cm above the eyebrows and adjusted using elastic bands on the head to ensure the middle detector was right above the FPz channel.

The experimental environment was in a soundproof room where participants sat comfortably facing a computer screen at a distance of 75 cm. A team member ensured accurate placement of the fNIRS headband on the participant’s head and adjusted it for comfort and good contact between the detectors and the scalp. All channels were checked for signal quality before the experiment began to ensure they met experimental requirements. Participants were instructed to remain still during the experiment.

The experimental content was displayed on a screen with a refresh rate of 60 Hz and a resolution of 2560 × 1440 pixels. Instructions were shown on the screen outlining the experimental process and the requirements participants were expected to follow. Initially, participants sat quietly while their brain activity was measured using fNIRS. During this resting-state measurement, participants were instructed to remain still and avoid any cognitive activity. Throughout data acquisition, participants maintained a comfortable and stable sitting posture, with hands placed naturally on their knees or the armrests of the chair. They were asked to fixate on a ‘+’ at the center of the screen, avoid looking around or engaging in conversation, and minimize body and head movements—including but not limited to raising eyebrows, frowning, moving ears, or scratching the head. After 5 minutes, the screen prompted participants to repeatedly recite the number sequence ‘1, 2, 3, 4, 5’ for 30 s. Subsequently, the screen instructed participants to begin a three-minute impromptu narration of their traumatic event experience, focusing on the worst event as defined by the CAPS-5 (Weathers et al., 2015). Finally, the screen again prompted participants to recite the number sequence ‘1, 2, 3, 4, 5’ for another 30 s. The narration of the traumatic event was synchronized with audio recording using a wired microphone connected to the computer, positioned 10–20 cm from the source of sound.

### Sociodemographic and clinical data processing

Sociodemographic and clinical variables were compared between PTSD and TEC groups. Data normality was assessed using the Shapiro–Wilk test (Shapiro & Wilk, 1965). Statistical analyses were performed using Pathon’s SciPy and pandas libraries. For continuous variables, statistical tests were selected based on data normality: independent samples *t*-tests were conducted for normally distributed data, and Mann–Whitney *U* tests were used for non-normally distributed data. Categorical variables were analyzed using Pearson’s Chi-square (*χ*^2^) test.

### Language data processing

Audio recordings were processed and analyzed using Python’s pydub library and librosa for acoustic analysis (Table 2). Google Cloud Speech-to-Text API was used to convert recordings into text accurately and generate markers for each word. The Chinese transcription text was processed using the jieba for segmentation. SpaCy was used for part-of-speech tagging and syntactic analysis to compute related linguistic indices (Table 2).

The pre-training phase for emotion analysis used a corpus containing 750MB of text from several major Chinese mental health forums (Li et al., 2024). This corpus was used to train word vectors through the large window WordVec algorithm, initializing the model’s word embedding layer. The model featured a hierarchical bi-directional recurrent neural network (biRNN) structure. The embedding vectors of each word are processed through the biRNN, where the forward RNN ( 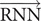 ) and backward RNN ( 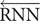 ) capture semantic dependencies from left to right and right to left, respectively. The forward and backward hidden states ( 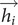 and 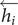 ) of each word are calculated and concatenated into 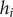 . For long texts, sentence-level segmentation is conducted, with each sentence 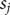 represented by its sequence of word embedding vectors processed through the same biRNN to produce a sentence representation 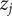 . The sequence of output vectors 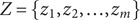 is then fed into an upper-level RNN ( 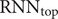 ), which generates an overall representation of the text. The final output layer of the model has been redesigned to include two parallel softmax layers. These layers predict the presence of emotional and factual vocabulary within the text while retaining marker information associated with each unit. The formula is expressed as follows:

Regarding the input sequence 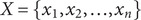 , each word 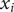 is transformed into a vector 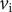 using the word embedding matrix 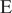 , and the entire sequence’s hidden states are then computed through the biRNN.
(1)

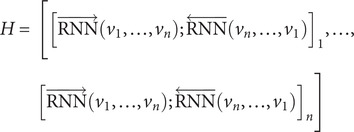


*H* is the output sequence processed by the biRNN.
(2)

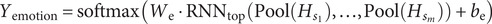



(3)

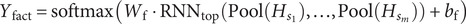


*Y* represents the final classification outputs, distinguishing between emotional and factual vocabulary. Further, this study utilizes the Linguistic Inquiry and Word Count (LIWC) to categorize types of emotions, feeding *Y*_emotion_ into LIWC2015 (McCarthy & Boonthum-Denecke, 2012) to ascertain the proportion of positive and negative emotional vocabulary within the text.

Data processing was performed using NumPy and pandas, and normality tests were conducted using SciPy. Group differences in linguistic features and vocabulary usage were analyzed using appropriate statistical tests based on data normality, as described above. Correlations between the frequency of emotional vocabulary and CAPS-5 symptoms were assessed using Pearson correlation coefficients (stats.pearsonr) for normally distributed data and Spearman’s rank correlation coefficients (stats.spearmanr) for non-normally distributed data.

### fNIRS data processing

fNIRS data were preprocessed using NirSpark software. Steps included motion artifact correction, removal of environmental and physiological noise with a 0.01–0.20 Hz bandpass filter, and conversion of optical density values using the modified Beer–Lambert law to compute changes in concentrations of oxyhemoglobin (Oxy-Hb) and deoxyhemoglobin (Deoxy-Hb).

Resting-state data were used to calculate the mean and standard deviation for each channel, and Z-score corrections were applied to data from the standard digit repetition and traumatic event narrative tasks. Markers from the onset of each negative emotional text unit were used to compute mean Oxy-Hb concentration changes within 10 s after the onset, subtracted by the average activation level during digit repetition tasks to determine changes relative to control conditions. Shapiro–Wilk tests assessed data normality. Differences in activation levels between PTSD and TEC groups during traumatic recall were analyzed using Mann–Whitney *U* tests, with p-values adjusted for false discovery rate (FDR).

Following the between-group comparisons, we extracted channel 27 (CH27), which showed the most significant intergroup difference, as region-of-interest feature variable for in-depth analysis. Precise cortical mapping was performed using the MPFC-ROIs^1^ tool, which identified the corresponding cortical region as the left amPFC (L-amPFC) (Lieberman et al., 2019). To investigate the temporal relationship between CH27 activation and negative emotional processing, we employed Spearman’s rank correlation coefficient in a time-series analysis.

## Results

### Demographic characteristics

As shown in Table 1, the study included 73 subjects: 35 diagnosed with PTSD according to DSM-5 criteria and 37 TEC. There were no significant differences between groups in terms of age, gender, or educational level. The average duration of illness among PTSD patients was 7.59 (SD = 5.97) years. Clinical evaluations using the CAPS-5 scale showed that the mean score for PTSD patients was 50.89 (SD = 7.57).

**Table 1.** Demographic characteristics of the study sample

Variables	PTSD patients	TEC	Effect sizes	*p*-value
N	35	37		
Age (years), mean (SD)^a^	28.49 (9.88)	29.70 (7.88)	0.077	0.567
Gender, *n* (%) female^b^	27 (77.14)	23 (62.16)	0.348	0.261
Years of education, mean (SD)^a^	15.19 (2.48)	13.92 (3.27)	0.243	0.078
Time since trauma (years), mean (SD)^a^	7.59 (4.98)	6.64 (3.20)	0.128	0.463
Trauma history, *n* (%)				
Physical abuse^b^	19 (48.72)	17 (46)	0.212	0.809
Sexual assault^b^	7 (17.95)	3 (8)	0.350	0.205
Witnessing someone else’s death^b^	9 (23.08)	12 (32)	0.146	0.362
Victim of violent criminal activity^b^	4 (10.26)	5 (14)	0.062	0.661
CAPS–5 scores, mean (SD)				
Intrusion symptoms^a^	12.77 (3.15)	0.89 (1.39)	0.998	<0.001**
Avoidance symptoms^a^	5.80 (1.59)	0.61 (1.34)	0.969	<0.001**
Cognitive and mood symptoms^a^	18.71 (2.72)	1.33 (1.72)	0.999	<0.001**
Arousal and reactivity symptoms^a^	13.60 (3.39)	1.44 (1.48)	0.999	<0.001**
Total^a^	50.89 (7.57)	4.28 (2.54)	0.999	<0.001**

N sample size SD standard deviation PTSD post-traumatic stress disorder TEC trauma-exposed controls CAPS-5 Clinician-Administered PTSD Scale for DSM-5 ** Indicates significance at the level of *α* = 0.01.

a Variables not following a normal distribution were analyzed using Mann–Whitney *U* tests; effect sizes are reported as the absolute value of Cliff’s delta.

b Categorical variables were analyzed using chi-squared tests; effect sizes are reported as the absolute value of Cohen’s h.

**Table 2.** Description of speech and language variables

Variables	Definition/calculation
Acoustic variables	
Pitch	Average fundamental frequency of speech measured in Hertz (Hz), indicating the overall highness or lowness of the voice
Pitch CV (%)	Coefficient of Variation of pitch (standard deviation divided by mean)
Energy	Average acoustic power of the speech signal
Energy CV (%)	Coefficient of Variation of energy
Semi-tone	Measures variations in pitch over time in semi-tones, capturing relative changes in pitch (intonation) rather than absolute frequency – highlighting differences from pitch by focusing on pitch fluctuations
Dynamic range	Difference between the maximum and minimum sound pressure level
RMS energy	Root Mean Square energy, a measure of the average power of a speech signal
Voiced segment duration	Average duration of segments containing voiced sounds
Pause count	Number of pauses
Pause mean duration	Average duration of pauses
Pause sd duration	Standard deviation of pause durations
Transcriptional variables	
Total number of words	Total words spoken in the analyzed speech
Articulation rate	Number of syllables/total phonation time
Mean length of utterance*	Average length of sentence
Type-token ratio	Number of types/number of tokens
Herdan’s C	Complexity measure based on the logarithmic ratio of types to tokens
Honoré’s R	Estimate of vocabulary richness, calculated from the total number of words and the number of words used only once, reflecting lexical diversity
Clauses per utterance*	Mean number of clauses per utterance
Noun-verb ratio	Number of nouns/number of verbs
Open-closed ratio	Number of open class words/number of closed class words
Percentage of disfluencies	Percentage of strange sentences
Char repetitiveness ratio (%)	Percentage of repeated characters in speech
Bigram repetitiveness (%)	Percentage of repeated two-character combinations
Trigram repetitiveness (%)	Percentage of repeated three-character combinations

* indicated the computation of the feature has been slightly adjusted to accommodate the differences in grammar structures between Chinese and English.

### Acoustic and transcription text features

Acoustic analysis of recordings from both the PTSD and control groups (Table 3) revealed significant differences in semitone value (*P* = 0.001), average pitch (*P* = 0.033), and average number of pauses (*P* = 0.040). Specifically, Both groups exhibited negative semitone values, indicating a downward trend in pitch, with a more pronounced decrease in the PTSD group (*M* = −15.11, SD = 6.53) than the controls (*M* = −17.10, SD = 6.59). PTSD patients had a higher average pitch (*M* = 201.21 Hz, SD = 32.84) compared with controls (*M* = 180.47 Hz, SD = 43.91). PTSD patients (*M* = 385.09, SD = 118.34) also had slightly more pauses in their narratives compared with controls (*M* = 362.60, SD = 316.22).

**Table 3.** Speech and language characteristics between groups

Variables	PTSD patients	TEC	Effect sizes	*p*-value
*N*	35	37		
Acoustic variables, mean (SD)				
Pitch^a^	201.31 (32.84)	180.47 (43.91)	0.536	0.033*
Pitch CV (%)^b^	16.31	24.33	—	—
Energy^a^	55.73 (5.72)	43.91 (7.37)	0.110	0.559
Energy CV (%)^b^	10.26	13.39	—	—
Semi-tone^a^	−15.11 (6.53)	−17.10 (6.59)	0.502	0.001**
Dynamic range^c^	0.29 (0.18)	0.30 (0.21)	0.001	0.976
RMS energy^c^	0.009 (0.01)	0.010 (0.02)	0.046	0.759
Voiced segment duration	68.82 (28.75)	58.47 (29.19)	0.085	0.188
Pause count^c^	385.09 (118.34)	362.60 (316.22)	0.289	0.040*
Pause mean duration^c^	0.34 (0.18)	0.53 (0.62)	0.053	0.714
Pause sd duration^c^	0.95 (0.77)	1.52 (3.04)	0.020	0.890
Transcriptional variables, mean (SD)				
Total number of words^b^	8581	7552	—	—
Articulation rate^c^	79.45 (36.24)	69.93 (31.66)	0.001	0.502
Mean length of utterance^a^	229.26 (113.11)	209.78 (93.66)	0.188	0.748
Type-token ratio^c^	0.56 (0.09)	0.56 (0.08)	0.036	0.780
Herdan’s C^c^	11.55 (58.29)	11.54 (28.29)	0.093	0.807
Honoré’s R^a^	30.23 (8.73)	34.59 (8.33)	0.510	0.032*
Clauses per utterance^c^	1.38 (1.23)	1.38 (0.73)	0.081	0.420
Noun-verb ratio^c^	0.66 (0.26)	0.63 (0.24)	0.089	0.525
Open-closed ratio^c^	1.81 (0.66)	2.16 (1.31)	0.135	0.324
Percentage of disfluencies^c^	0.06 (0.04)	0.06 (0.02)	0.080	0.566
Char repetitiveness ratio (%)^b^	73.33	76.85	—	—
Bigram repetitiveness (%)^b^	29.56	33.08	—	—
Trigram repetitiveness (%)^b^	10.80	6.56	—	—
Emotional variables, mean (SD)				
Logical fact vocabulary^c^	2.35 (1.32)	2.67 (1.71)	0.051	0.664
Positive emotion^c^	1.30 (0.48)	1.73 (0.65)	0.183	0.120
Negative emotion^c^	5.89 (3.00)	4.21 (2.83)	0.339	0.021*

N sample size SD standards deviation * Indicates significance at the level of *α* = 0.05. ** Indicates significance at the level of *α* = 0.01.

a Variables following a normal distribution were analyzed using *t*-tests; effect sizes are reported as the absolute value of Cohen’s d.

b Not included in the group differences test, only displayed for referential purposes.

c Variables not following a normal distribution were analyzed using Mann–Whitney *U* tests; effect sizes are reported as the absolute value of Cliff’s delta.

Analysis of transcriptions (Table 3) showed a significant difference in Honoré’s R, which reflects lexical richness. The PTSD group had a lower richness in vocabulary usage (*M* = 30.23, SD = 8.73) compared with controls (*M* = 34.59, SD = 8.33; *P* = 0.032). There were no significant differences between the groups in other linguistic forms and structural features.

### Language emotional variables

Comparative analysis of language-emotional variables between PTSD patients and controls (Table 3) revealed no significant differences in the use of factual vocabulary. However, analysis of emotional vocabulary indicated that PTSD patients (*M* = 5.89, SD = 3.00) used negative emotional words significantly more frequently compared with the control group (*M* = 4.21, SD = 2.83; *P* = 0.021). There was no significant difference in the use of positive emotional words, though the frequency was slightly higher in the control group (*P* = 0.372).

Additionally, statistical analysis of the correlation between the frequency of negative emotional words and CAPS-5 scores in PTSD patients and controls was conducted. There was a significant correlation between the total scores of cognitive and mood symptoms under criterion D of the CAPS-5 and the frequency of negative emotional words (*r* = 0.349, *P* = 0.037).

### Near-infrared brain activation patterns

**Figure 1.**
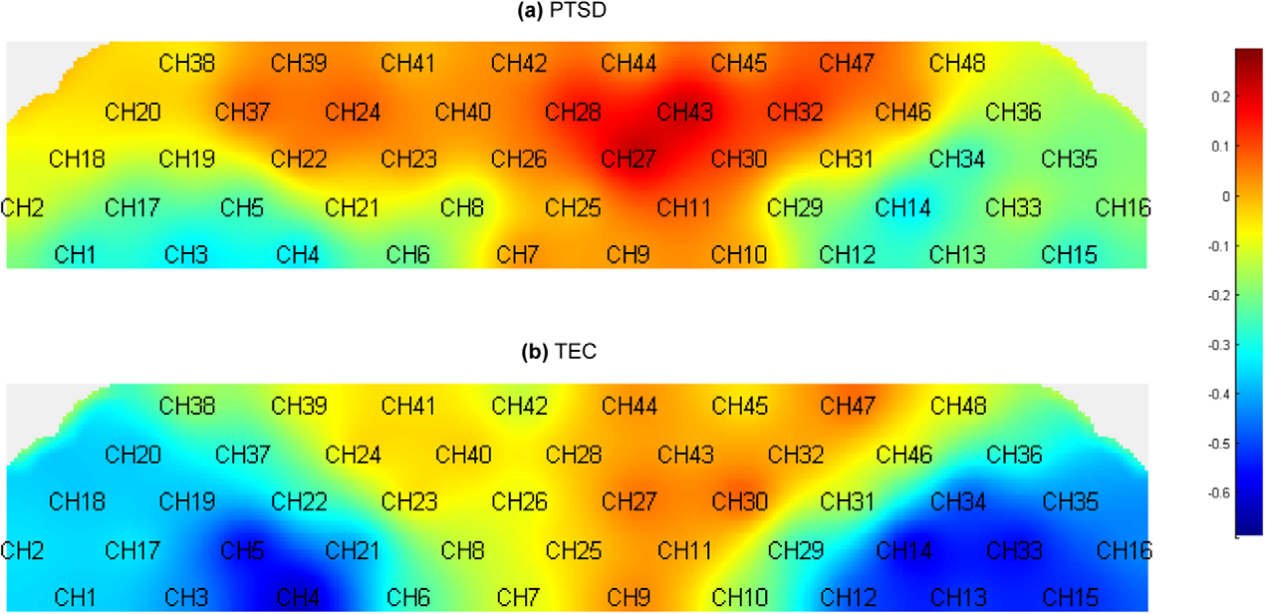
Activation Map of Negative Emotional Memory Words in 2D. The chart indicates the location of leads on the fNIRS cap, with MNI coordinates for CH27: x = -15.37, y = 72.29, z = 11.12. (a) Average activation map in a 10-second window following the extraction of negative emotional memory words during the traumatic event narration task in the PTSD group. (b) Average activation map in a 10-second window following the extraction of negative emotional memory words during the traumatic event narration task in the TEC (trauma-exposed controls) group.

**Figure 2.**
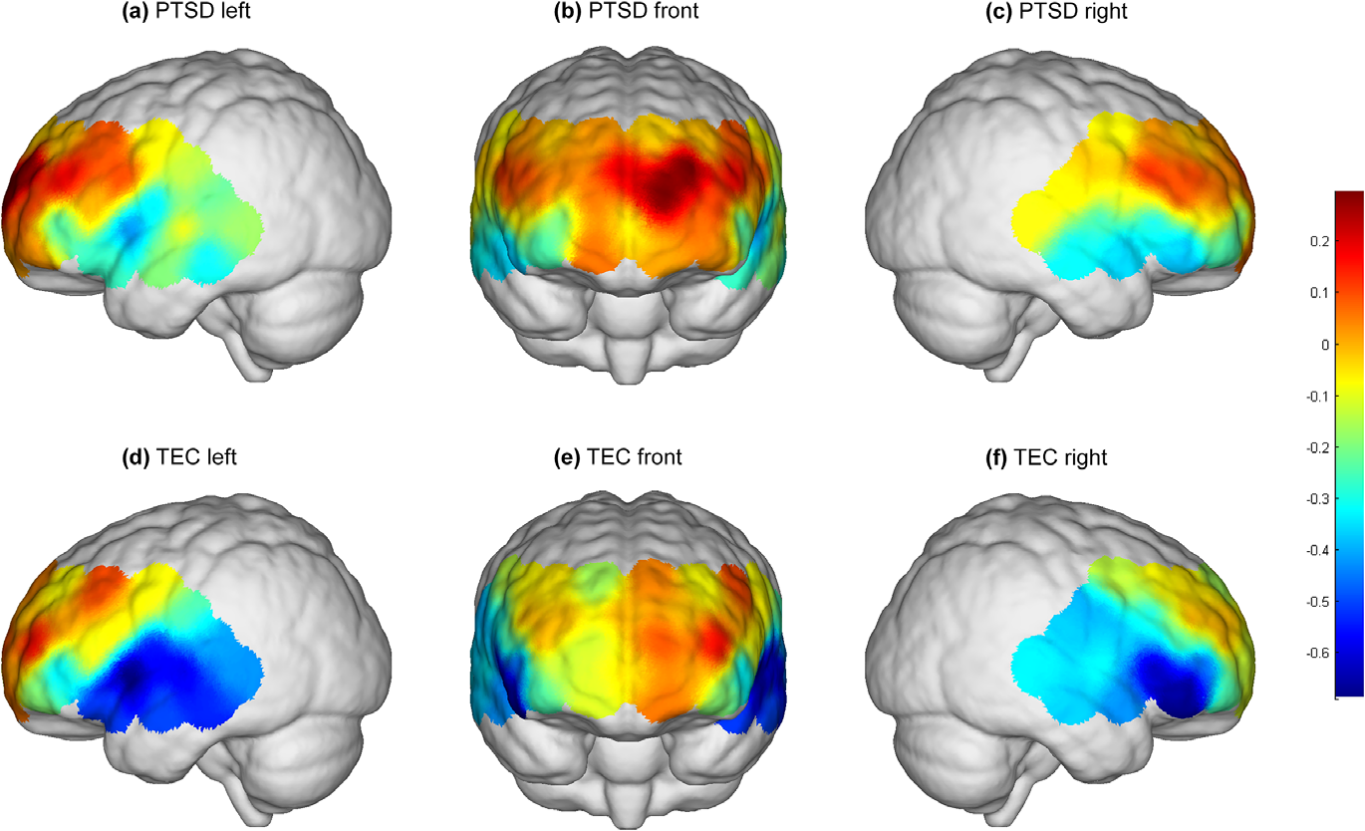
3D Activation Map of Negative Emotional Memory Words. Within a 10-second window following the appearance of negative emotional words, the PTSD patients showed higher activation levels (mean Oxy-Hb activation = 0.295) in the CH27 region of the left anterior medial prefrontal cortex compared to the control group (mean Oxy-Hb activation = 0.086). This difference was statistically significant after FDR correction (p < 0.001).

**Figure 3.**
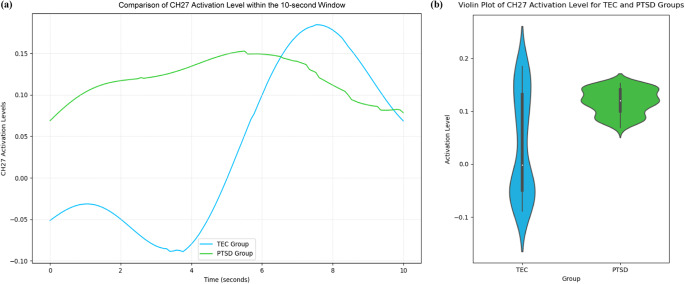
Correlation of CH27 Activation Levels with Negative Affective Memory Vocabulary. (a) Time-Series Analysis of CH27 Oxy-Hb Activation Levels in TEC and PTSD Groups. Within the 10s window after negative emotional vocabulary appearance, the TEC group showed a distinct upward and then downward trend starting at 3.82s, while the PTSD group remained at a higher level of activation. (b) Distribution of CH27 Oxy-Hb Activation Levels in TEC and PTSD Groups.

Oxy-Hb values following the expression of negative emotional words in the PTSD and TEC groups showed significant differences within the 10 s window. Results from Figures 1 and 2 indicated that, at CH27, located in the L-amPFC (Lieberman et al., 2019), PTSD patients exhibited higher levels of Oxy-Hb activation compared with controls, with statistically significant differences after FDR correction (*p* < 0.001). Specifically, the activation level in CH27 for the PTSD group remained elevated (*y*_max_ = 0.153, *y*_difference_ = 0.084) with no apparent latency period. Although there was a slight fluctuation in activation levels during this period, there was no significant correlation with time (Spearman’s *r* = 0.026, *p* = 0.785) (Figure 3).

In contrast, the TEC group showed a statistically significant positive correlation with time under the same conditions (Spearman’s *r* = 0.727, *p* < 0.01). At 3.82 s after using negative affective vocabulary, the CH27 region Oxy-Hb saturation in the TEC group began to rise significantly, reaching a peak 3.73 s later (*y*_max_ = 0.184, *y*_difference_ = 0.273). The rate of change in activation level was approximately 0.0067, followed by a downward trend. There were significant fluctuations in CH27 activation levels in the TEC group, reflecting notable changes in activation levels within the time window following the expression of negative affective vocabulary.

## Discussion

This study aimed to investigate the relationships among linguistic features, traumatic fear memory, and activation of the amPFC in PTSD patients compared with a control group. Our findings reveal three major insights: (1) PTSD patients relied more on explicit narratives of negative emotional memories during traumatic recall and experienced greater emotional distress; (2) both healthy controls and PTSD patients exhibited activation of the L-amPFC following the expression of negative emotions; however, activation in healthy controls showed significant temporal sequence correlations. In contrast, (3) PTSD patients showed sustained high activation levels in the L-amPFC, particularly during the expression of negative emotional words, contrasting sharply with the control group’s rapid and effective emotional regulation capabilities, suggesting an overactivity of the low pathway (rapid emotional responses) and insufficient regulation of the high pathway (detailed cognitive processing).

The results indicate that PTSD patients depend more on explicit narratives of negative emotional memories and experience heightened emotional distress during their expression. Acoustic analysis showed that PTSD patients had a significantly higher pitch, potentially linked to anxiety or tension while recalling traumatic events. Furthermore, the analysis of semitone values suggested that PTSD patients exhibited less pitch decline, reflecting a more monotonic and less varied tone during their traumatic narrations, possibly associated with numbing or subdued effect. This is consistent with previous findings indicating a more monotonous voice in PTSD patients (Marmar et al., 2019). Additionally, PTSD patients exhibited more frequent pauses during their narratives, likely due to greater emotional distress or difficulty in organizing language while recalling and narrating traumatic events, also suggesting that their recollections are not merely rote descriptions.

Analysis of transcriptions revealed a lower Honoré’s R index in PTSD patients, indicating reduced complexity and diversity in their vocabulary during traumatic narrations. However, no significant differences were found in other linguistic structural features between the PTSD and control groups, affirming that PTSD primarily manifests as changes in emotional, cognitive, and physiological responses. While these may affect the emotional tone and content of language expression, they do not significantly alter its structural characteristics. This contrasts with other mental disorders such as schizophrenia, which can lead to significant changes in language structure, such as formal thought disorder (De Boer et al., 2020; Li et al., 2024). The linguistic characteristics of PTSD patients tend to manifest more in terms of affect and content rather than form and structure.

Furthermore, the analysis of the use of emotional and factual vocabulary revealed that PTSD patients described factual and logical information similarly to the control group. However, upon further subdivision of emotional vocabulary, this study found that PTSD patients more frequently used negative emotional words in their language, which may suggest that they experience stronger negative emotions when recounting traumatic experiences. Given that this study did not directly measure negative emotions, this interpretation has certain limitations and should be considered speculative. Nevertheless, this finding aligns with one of the core symptoms of PTSD – the negative alterations in cognition and mood (Krüger-Gottschalk et al., 2022).

In learning and memory, there is a process where short-term memories stored primarily in the hippocampus and amygdala are transformed into long-term memories stored in the prefrontal cortex (Frankland & Bontempi, 2005; Kitamura et al., 2017). Memory can be disrupted and destroyed through reconsolidation processes (Lacagnina et al., 2019; Ressler et al., 2021). Research by Frankland and Bontempi has shown that the mPFC plays a crucial role in maintaining long-term fear memories (Frankland & Bontempi, 2005). The transition from acquiring short-term memory to storing it as long-term memory involves a complex and slow process of enhancing and establishing cortical connections, and the mechanisms for storing and maintaining long-term memory remain unclear.

Our study, by monitoring brain activity during descriptions of negative emotional states, found significant differences in brain activity between PTSD patients and the control group. Notably, within a 10 s window after the expression of negative emotional words, PTSD patients maintained higher activation levels in the L-amPFC, suggesting potential regulatory issues in suppressing or diminishing fear memories. In contrast, the control group’s activation levels showed a significant positive correlation with time, indicating their more effective handling and mitigation of negative memory impacts.

These findings are consistent with prior studies on the role of the prefrontal-amygdala network in the consolidation of fear memories. During the arousal of fear memories, significant neuronal firing activity in the rat infralimbic (IL) region was negatively correlated with the intensity of fear responses. Additionally, the process of fear memory extinction further enhanced activity levels in the IL region. When a fear stimulus was presented, increased activity in the IL region played an inhibitory role, thereby reducing the expression of fear responses (Ashokan et al., 2018; Awad et al., 2015; Bloodgood et al., 2018). Research by Neumeister et al. (2017) found significant increases in anterior cingulate cortex (ACC)/mPFC activation in PTSD patients when exposed to traumatic cues, with hyperconnectivity between the BLA and ACC/mPFC, playing a crucial role in maintaining fear memories, particularly pathological memories. This hyperconnectivity might prevent normal suppression or delay in the diminishment of fear memories in PTSD patients, explaining the sustained high activation levels in the L-amPFC observed in our study, in contrast to the linear decrease observed in the control group after reaching a peak.

During the process of fear memory extinction, as cues associated with fear in the environment change, the expression of conditioned fear memories is suppressed, forming new extinction memories (Bouton et al., 2021; Herry et al., 2010). These extinction memories involve the same brain regions as fear memory acquisition, storage, and retrieval but engage different neuronal circuits. The brainstem and BLA play a primary role in the early formation of extinction memories, with the consolidation of these memories requiring involvement from the IL region and a reduction in activity in the PrL region (Bouton et al., 2006; Burgos-Robles et al., 2009; Klavir et al., 2017; Quirk & Mueller, 2008), while the hippocampal–cortical circuit mediates the rekindling of fear memories (Bernier et al., 2017; Campese & Delamater, 2014; Maren et al., 2013). Our study found that after describing traumatic experiences using negative emotional words, the L-amPFC region’s oxygen saturation in the TEC group began to significantly rise and then showed a downward trend, possibly reflecting a reduction in fear information encoding and subsequent fear memory extinction. This aligns with previous research on extinction memory mechanisms and further supports the dynamic interplay between extinction and fear memories.

The time-series results from our study support the dual-pathway model of emotional responses to fear-inducing stimuli, which posits the existence and functionality of both low and high pathways. Our findings reveal that individuals in the control group displayed rapid and effective emotional regulation when confronted with negative emotional words, characterized by a quick rise and fall in L-amPFC activity. This phenomenon aligns with the role of the high pathway in emotional regulation, which involves more detailed emotional analysis and regulation to maintain emotional stability. Conversely, PTSD patients exhibited a sustained high activation state under the same conditions, likely indicating an overactivity of the low pathway. Given the rapid response and lower precision of the low pathway, it may lead to an exaggerated response to negative stimuli in PTSD patients, where the regulatory functions of the high pathway fail to intervene effectively, resulting in emotional regulation difficulties. These findings suggest that emotional regulation issues in PTSD may stem from a functional imbalance between the two pathways, rather than issues within a single pathway.

Consequently, our results provide new insights for future therapeutic strategies. In treating PTSD, there should be a particular focus on enhancing the functionality of the high pathway to improve patients’ emotional regulation capabilities. This may include techniques such as cognitive-behavioral therapy to strengthen cognitive processing capabilities toward negative emotional stimuli, reducing reliance on the low pathway and thus improving the adaptability and stability of emotional responses.

## Footnotes

1
https://github.com/MetaD/MPFC-ROIs
